# Mapping dengue risk in Singapore using Random Forest

**DOI:** 10.1371/journal.pntd.0006587

**Published:** 2018-06-18

**Authors:** Janet Ong, Xu Liu, Jayanthi Rajarethinam, Suet Yheng Kok, Shaohong Liang, Choon Siang Tang, Alex R. Cook, Lee Ching Ng, Grace Yap

**Affiliations:** 1 Environmental Health Institute, National Environment Agency, Singapore; 2 Environmental Public Health Operations, National Environment Agency, Singapore; 3 Saw Swee Hock School of Public Health, National University of Singapore and National University Health System, Singapore; 4 School of Biological Sciences, Nanyang Technological University, Singapore; Institute for Disease Modeling, UNITED STATES

## Abstract

**Background:**

Singapore experiences endemic dengue, with 2013 being the largest outbreak year known to date, culminating in 22,170 cases. Given the limited resources available, and that vector control is the key approach for prevention in Singapore, it is important that public health professionals know where resources should be invested in. This study aims to stratify the spatial risk of dengue transmission in Singapore for effective deployment of resources.

**Methodology/principal findings:**

Random Forest was used to predict the risk rank of dengue transmission in 1km^2^ grids, with dengue, population, entomological and environmental data. The predicted risk ranks are categorized and mapped to four color-coded risk groups for easy operation application. The risk maps were evaluated with dengue case and cluster data. Risk maps produced by Random Forest have high accuracy. More than 80% of the observed risk ranks fell within the 80% prediction interval. The observed and predicted risk ranks were highly correlated (ρ ≥0.86, P <0.01). Furthermore, the predicted risk levels were in excellent agreement with case density, a weighted Kappa coefficient of more than 0.80 (P <0.01). Close to 90% of the dengue clusters occur in high risk areas, and the odds of cluster forming in high risk areas were higher than in low risk areas.

**Conclusions:**

This study demonstrates the potential of Random Forest and its strong predictive capability in stratifying the spatial risk of dengue transmission in Singapore. Dengue risk map produced using Random Forest has high accuracy, and is a good surveillance tool to guide vector control operations.

## Author summary

Dengue fever, the most prevalent mosquito-borne viral disease today, is caused by Dengue virus (DENV) and transmitted to human by *Aedes* mosquitoes, primarily the *Ae*. *aegypti* and *Ae*. *albopictus*. The key approach to mitigating dengue transmission is to control the *Aedes* population, and this often involve vector control strategies such as larval source reduction and preventive surveillance that are labour-intensive and require effective deployment of valuable resources. Spatial risk profiling of dengue transmission is therefore necessary to ensure the optimal utilization of limited resources, and achieving maximum impact of dengue vector control. Here, we developed a dengue risk map by stratifying the spatial risk of dengue transmission in Singapore. Random Forest was used to predict the risk rank of dengue transmission in 1km^2^ grids, and the predicted risk ranks are then categorized and mapped to color-coded risk groups. The dengue risk map is a good surveillance tool to guide vector control operations. Valuable resources can be deployed in a strategic manner, mitigating the spread of dengue transmission.

## Introduction

Dengue is a viral infection caused by one of the four closely related yet antigenically distinct virus serotypes (DENV-1, DENV-2, DENV-3 and DENV-4), and transmitted by *Aedes* mosquitoes, primarily the *Ae*. *aegypti* and *Ae*. *albopictus* [1,2]. Infection confers lifelong immunity to the infecting serotype [3]. However, it increases risk for dengue haemorrhagic fever (DHF) and dengue shock syndrome (DSS), a deadly form that present with severe complications, in subsequent infections [4]. Since the publication of the GBD 2010, it was estimated that 390 million dengue infections occur each year globally, of which 500,000 develop into DHF [5,6]. Dengue poses a substantial public health threat globally, especially throughout the tropical and subtropical regions [7,8].

Located one and a half degrees north of the equator and lying in the dengue belt, Singapore is prone to dengue transmission, with all four dengue serotypes co-circulating and frequent introduction of new genotype virus [9]. Though intensive vector control efforts have successfully suppressed the *Aedes* population, from an *Aedes* house index of over 50% in the 1960’s to the present 1–2%, Singapore remains susceptible to dengue outbreaks [10–12]. The increased in human population density and the low herd immunity resulting from sustained period of low dengue transmission are factors that may have contributed to the resurgence of dengue in Singapore [13,14]. A significant amount of funding and resources has been allocated for dengue every year [15]. The estimated economic and disease burden of dengue were 9–14 disability-adjusted life years (DALYs) per 100,000 population and US$41.5 million per annum [16].

A dengue temporal model was developed in 2013 by the Environmental Health Institute, a research institute of the Singapore’s National Environment Agency (NEA) in collaboration with the National University of Singapore (NUS) to aid vector control measures. The model predicts trends and incidence up to 12 weeks ahead, providing early warnings of outbreak and facilitating public health response to moderate impending outbreak [17]. This model was able to accurately project an upward trend of dengue cases in 2013 and 2014, predicting the two major outbreaks [18]. NEA has been using the model in planning vector control and public communication [19]. However, a limitation of the model is the missing spatial resolution as it does not highlight areas with high risk of dengue transmission. Given that NEA’s key strategy in dengue control is preventive surveillance and larval source reduction, a labour-intensive activity that requires effective deployment of a limited pool of skilled vector control officers, spatial risk profiling of dengue transmission is thus necessary for effective deployment of resources, and achieving maximum impact.

In this paper, we describe a new approach for spatial risk stratification of dengue transmission in Singapore. Using Random Forest, we quantify the risk of dengue transmission in different areas and categorize them into different risk groups to guide the pre-emptive source reduction exercise conducted by NEA vector control officers. Predictive performance of the model is evaluated with both dengue cases and clusters.

## Materials and methods

### Statistical analysis

**Fig 1:**
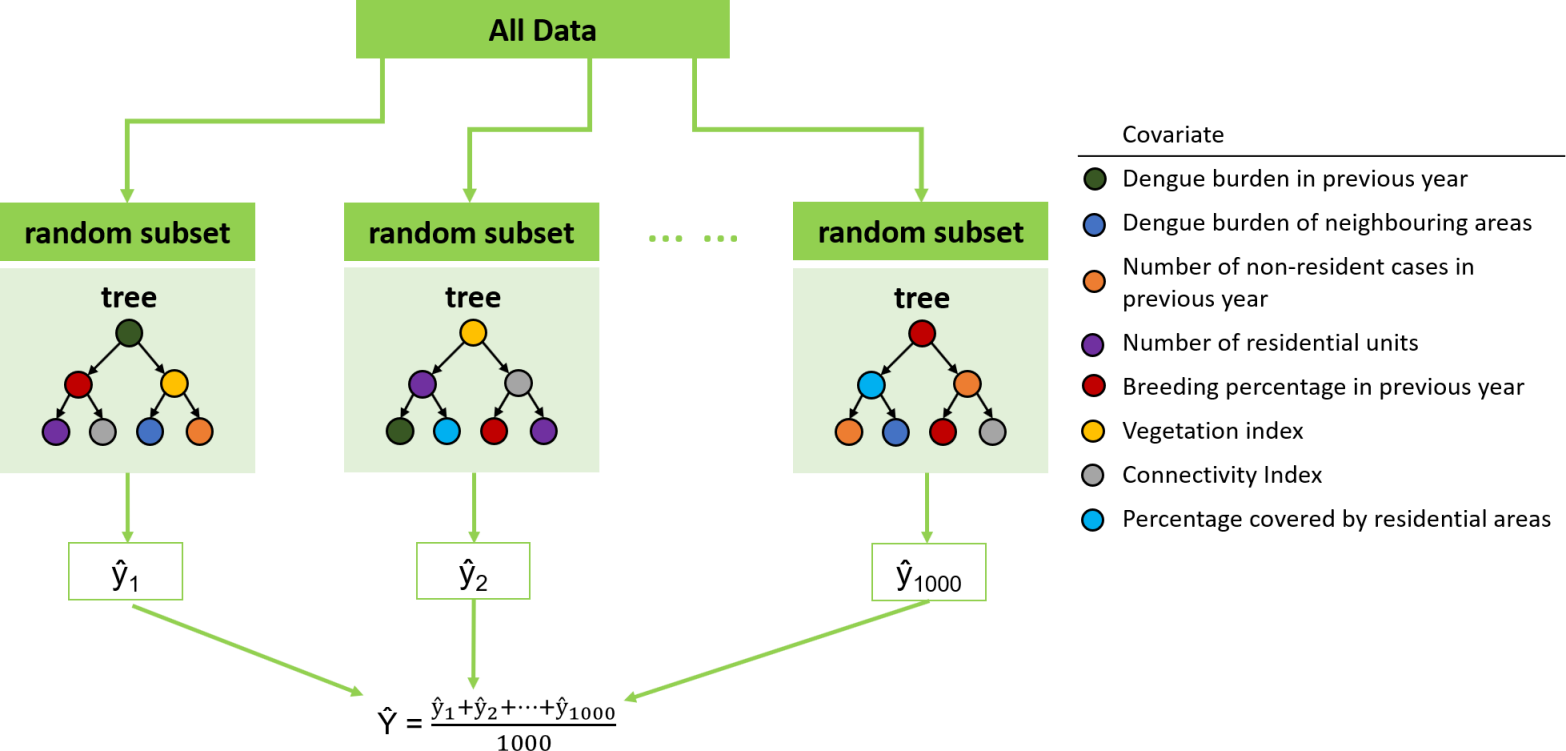
Framework of random forest algorithm. 1000 random bootstrap samples were drawn from the data, and an unpruned decision tree is fitted to each bootstrap sample. At each node, a small subset of the covariates was chosen at random to optimize the split. The predicted risk rank is obtained by averaging the prediction of all trees.

Proposed by Leo Breiman, Random Forest is an ensemble machine learning method that uses an ensemble of decision trees [20]. In Random Forest, several (N = 1000) bootstrap samples are drawn from the training set data, and an unpruned decision tree $f_{n}(x)$, is fitted to each bootstrap sample. At each node of the decision tree, variable selection is carried out on a small random subset of the predictor variables, so as to avoid the “small *n* large *p*” problem. The best split on these predictors is used to split the node. The predicted response is obtained by averaging the predictions of all trees, i.e. $\frac{1}{N}\sum_{n\;=\;1}^{N}f_{n}(x)$ (Fig 1). Random Forest was used to predict the percentile rank of dengue case count in 1km^2^ grids, with past dengue exposure (total number of cases in previous year, total number of cases in neighbouring grids in previous year and number of non-resident cases in previous year), human population (estimated population density), vector population (estimated ratio of *Aedes aegypti* mosquitoes out of all *Aedes* moquitoes—breeding percentage) and environmental data (vegetation index, connectivity index and ratio of residential area). The predicted percentile ranks are then categorized and mapped to four color-coded risk groups (RG1-4, lowest risk as RG1 and highest risk of dengue transmission as RG4) for easy operation application. Although administrative boundaries are more compatible with ground operation, 1km^2^ grids were used as study units as they are more consistent in area size and do not change over time. We use residential grids exclusively for the analysis and risk mapping. Random Forest analyses were performed using the randomForest package implemented in the R statistical language [21].

### Model evaluation

Data from 2006 to 2013 were used to parameterize the model, and performance of the model is evaluated with new dengue case data from 2014 to 2016. Apart from visually comparing the risk map and distribution of dengue cases, we applied the following quantitative metrics to evaluate the model: 1. correlation between predicted and observed percentile ranks, 2. coverage of prediction intervals, 3. summary statistics of the number of cases within each risk group, and 4. weighted (square) Kappa agreement coefficients of risk grouping.

In addition to using dengue case data, data on dengue cluster, which indicates possible transmission within the locality, were considered for model evaluation as well. We investigated the odds of clusters forming in high (RG 3 and 4) and low (RG 1 and 2) risk areas, and examined if transmission intensity, comprising of cluster’s growth rate, transmission duration and cluster size differ between high and low risk areas. Differences were analysed using Kruskal-Wallis tests.

### Data

Table 1 shows the various risk factors considered for the risk mapping. The risk factors were identified from literature review and examined with historical data [11,22,23]. All data (Dengue, Population and Entomological) were aggregated to the 1km^2^ grids. The time period used for all variables was January 2006 to December 2016, and their sources are:

**Table 1:** Overview of the risk factors used for dengue risk mapping.

Category	Type	Covariates
Dengue exposure	Spatio-temporal	Dengue burden in previous year
		Dengue burden of neighbouring grids in previous year
		Number of non-resident cases in previous year
Population	Spatio-temporal	Number of residential units
Entomological	Spatio-temporal	Breeding percentage in previous year
Environmental	Spatial	Vegetation index
	Spatial	Connectivity index
	Spatial	Percentage covered by residential areas

#### Dengue cases

Dengue is a notifiable disease in Singapore, where medical practitioners are required to notify all clinically diagnoses and laboratory confirmed dengue cases to the Ministry of Health (MOH), Singapore [24]. Residential and workplace address and onset date of each dengue cases are recorded and shared with NEA on a daily basis. Dengue cases were tagged to the address, either residential or workplace address, after epidemiological investigation has been carried out by officers to determine and confirm the location where the cases acquired dengue. The addresses were then geocoded using the Geographic Information System (GIS). Geo-referenced data on dengue cases was extracted from the GIS database of NEA, and anonymized prior to analysis.

#### Population density

The number of residential units were provided by Housing Development Board (HDB) for public housing and sourced online from the Real Estate Information System (REALIS), an online database managed by Urban Redevelopment Authority, for private housing.

#### Entomological

Breeding Percentage (BP) is an in-house index developed by NEA to estimate the proportion of *Ae. aegypti* relative to *Ae. albopictus*, which is ubiquitous in Singapore [25]. BP is calculated from the number of *Aedes* mosquito breeding sites recorded during ground inspections carried out by NEA using the formula:

$$BP=\frac{N_{aegypti}\left(t\right)}{N_{aegypti}\left(t\right)+N_{albopictus}\left(t\right)-N_{mixed}\left(t\right)}$$

NEA carried out routine inspection surveillance across Singapore throughout the year. These inspections include those scheduled for regular preventive surveillance, and those conducted in response to dengue transmission in a location. To estimate the yearly BP for each grid, geo-referenced data on *Aedes spp*. larval counts from the routine surveillance was extracted from the GIS database of NEA and mapped the location of *Aedes* breeding sites onto each grid to extract the number of *Ae*. *aegypti* and/or *Ae*. *albopictus* breeding sites found within each grid for each year. BP value for grids with inspections was calculated by definition. For grids that were not inspected, their BP values were estimated using ordinary Kriging with a spherical variogram model.

#### Environmental factors

Vegetation index, also known as the Normalized Difference Vegetation Index (NDVI), is an index of plant “greenness” or photosynthetic activity. NDVI data was provided by Centre for Remote Imaging, Sensing and Processing in NUS after processing satellite image. Connectivity index measures the total connectivity (accessibility) of the grid relative to all other grids, and is derived from public transport data from Future City Lab ETH-NUS.

#### Dengue clusters

Dengue cases are clustered for vector operations purposes based on their geographical and temporal proximity. A dengue cluster is formed when two or more cases are located within a 150-meter radius and with the onsets of illness within a 14-day period. Dengue clusters are generated using the Geographical Information System (GIS), and information such as transmission duration, serotypes detected and the number of dengue cases is recorded for every cluster [12].

## Results

**Fig 2:**
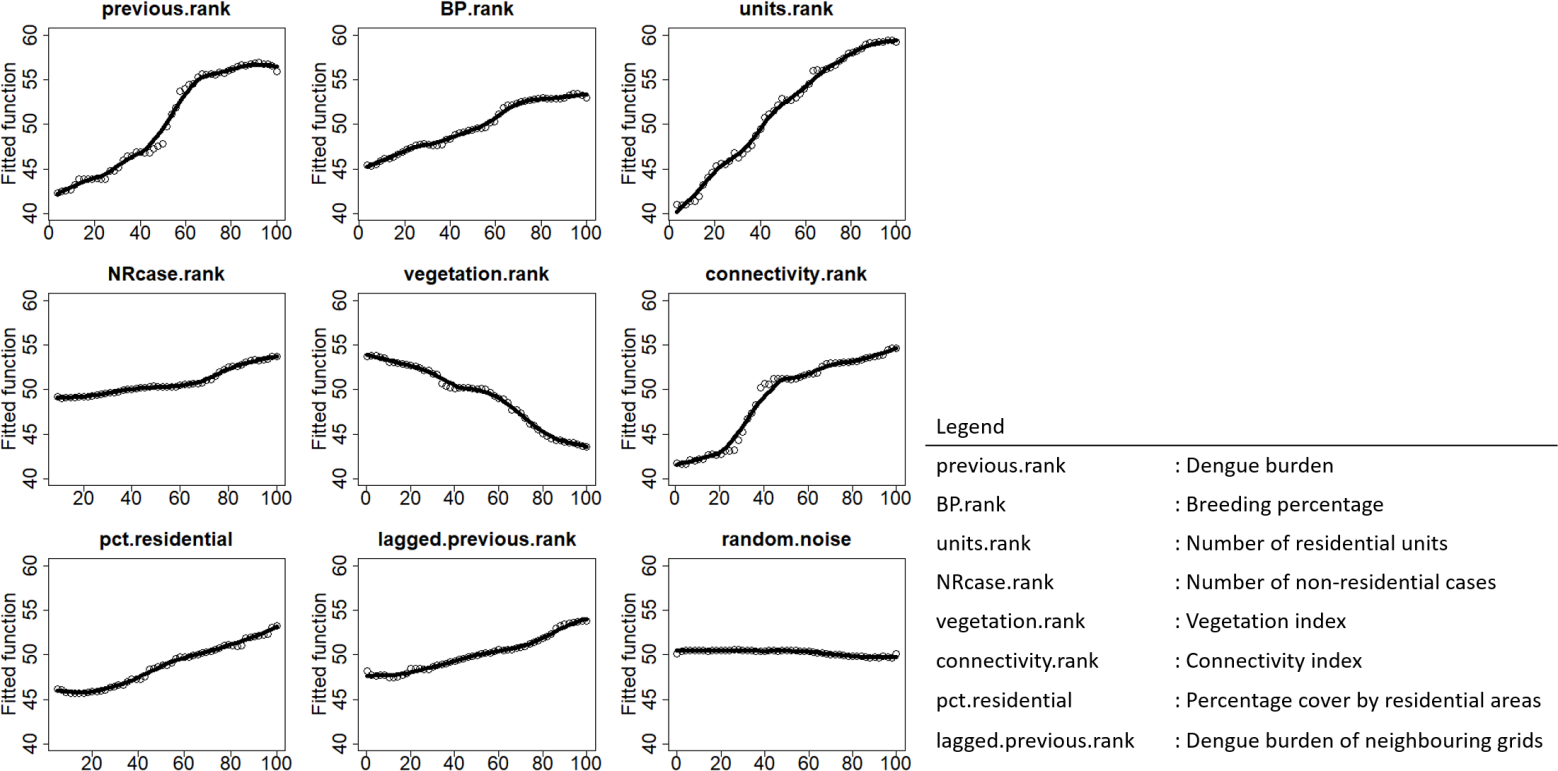
Partial dependence plot of the risk factors showing how dengue burden varies with one variable when all other variables are held constant at their average values.

**Fig 3:**
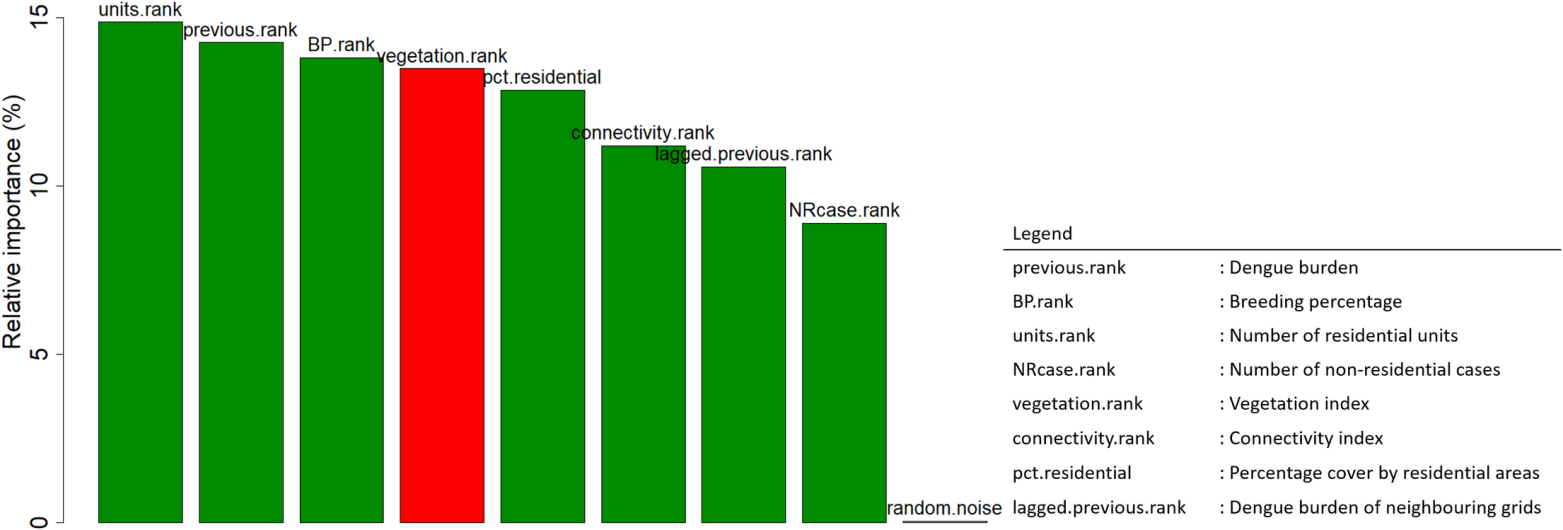
Variable importance plot showing population density, dengue burden and breeding percentage having stronger predictive power than other variables.

Associations between covariates and dengue burden were examined through partial dependence plot. Consistent with our prior knowledge, all covariates are associated with dengue burden, as contrasted by the flat line partial effect of random noise (Fig 2). Among the covariates, the number of residential units, dengue burden in previous year and the breeding percentage in previous year are top-ranked in terms of variable importance (Fig 3), and impose a larger influence on model accuracy, relative to the other covariates. This, therefore, suggests that population density, dengue burden and abundance of *Ae*. *aegypti* are significant risk factors for dengue transmission.

**Fig 4:**
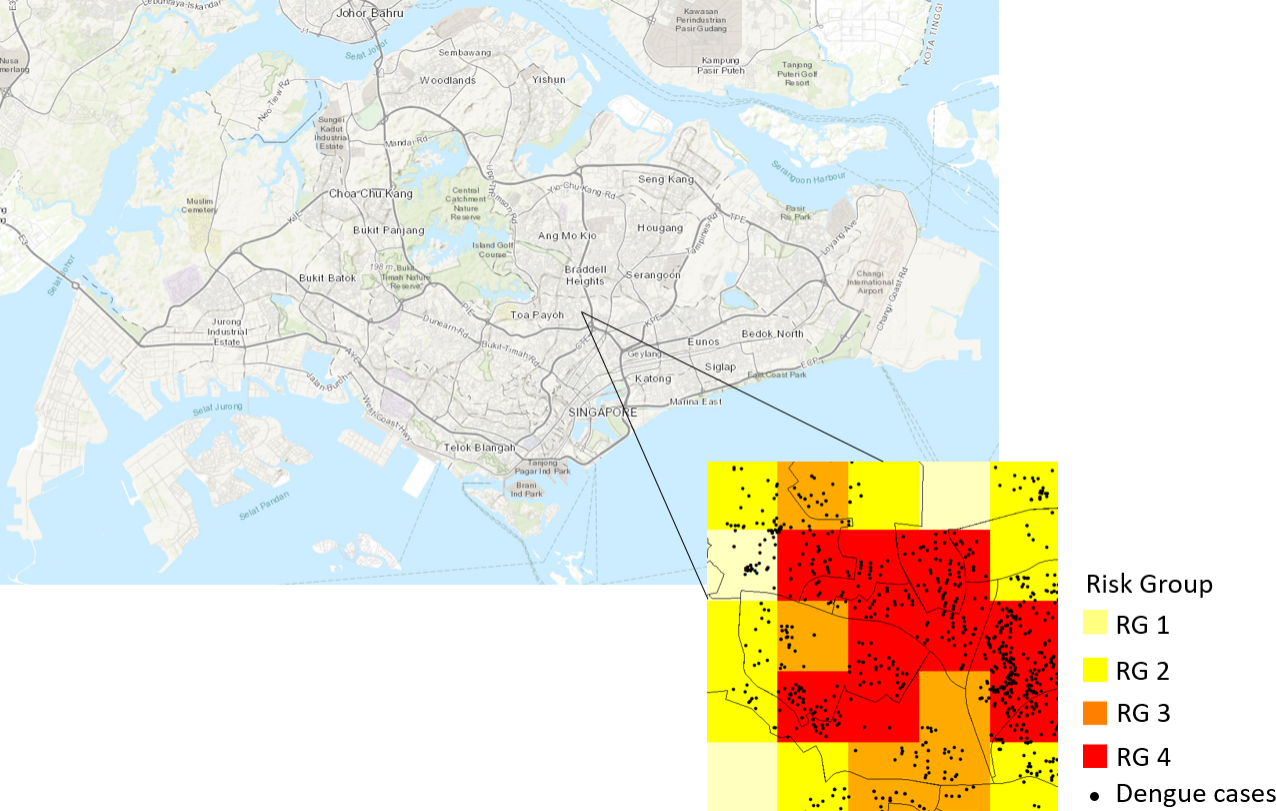
Risk grouping of a small area with 2016 dengue cases (black circles) overlaid. **The risk groups are color-coded, with RG 4 (highest risk) as red and RG 1 (lowest risk) in light yellow. The figure was created using R software with base layer obtained from https://landsatlook.usgs.gov/**.

**Fig 5:**
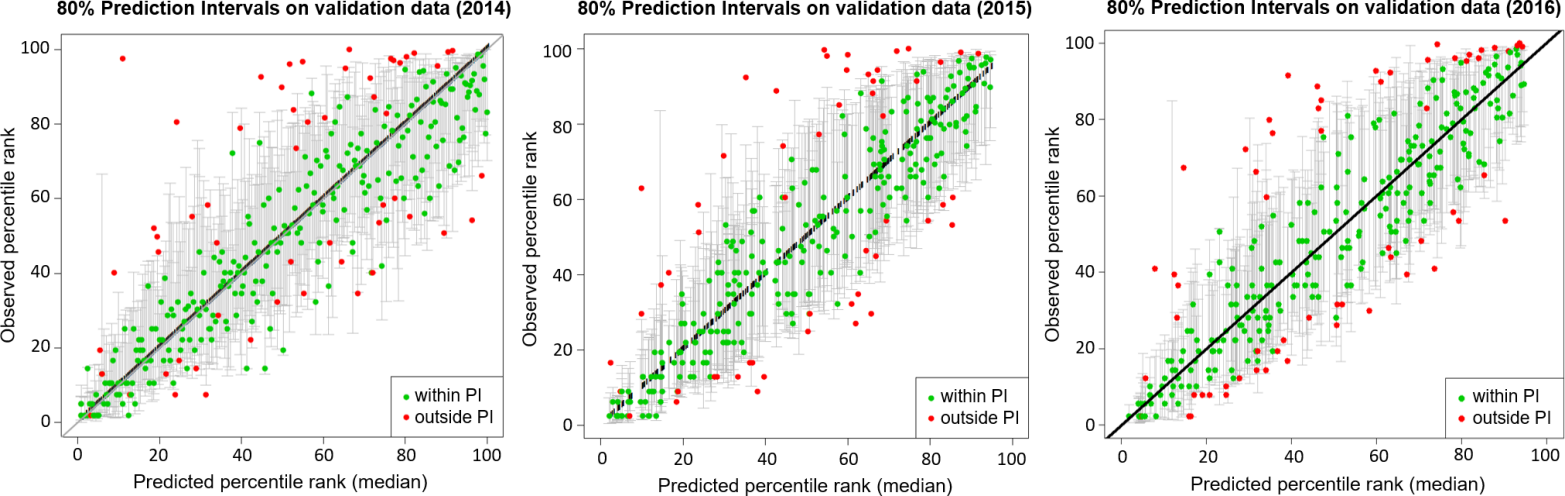
The predicted percentile ranks and its 80% prediction interval for 2014 to 2016 (left to right). In each panel, the green circles indicate the predicted percentile ranks that fall within the prediction intervals and the red circles indicate the predicted percentile ranks that fall outside the prediction intervals.

The predicted percentile ranks were categorized and mapped to four color-coded risk groups based on the three quartiles so that the number of grids in each risk group is approximately the same. The distribution of risk groups is comparable in all three years, with high risk groups (RG 3 and 4) congregating in the eastern part of Singapore. When dengue cases were overlaid onto the risk maps, we observed good agreement between the cases and risk groups (Fig 4). Majority of the cases fell in risk group 3 and 4. There was strong positive correlation between the observed and predicted risk ranks, a correlation of 0.86 (P <0.01), 0.87 (P <0.01) and 0.88 (P <0.01) in 2014, 2015 and 2016 respectively. In addition, the risk level commensurate with case density. The predicted risk levels were in excellent agreement with the case density, a weighted Kappa coefficient of 0.814 (P <0.01) in 2014, 0.839 (P <0.01) in 2015 and 0.821 (P <0.01) in 2016. This is further supported by the increasing trend of dengue case count from risk group 1 to 4 (Table 2). Fig 5 shows the predicted percentile ranks and its 80% prediction interval. 82% and 83% of the observed percentile ranks fell within the 80% prediction interval in 2014 and 2015 respectively. In 2016, 81% of the observed percentile ranks fell within the 80% prediction interval. Overall, cases in 2015 have slightly better agreement with the risk map than in 2014 and 2016.

**Table 2:** Summary statistics of dengue case count in grids for all risk groups in 2014–2016.

Year	Risk Group	Percentile Rank	Count	Min	1^st^ Quartile	Median	Mean	3^rd^ Quartile	Max
2014	1	[0, 25)	79	0.0	2.0	4.0	8.5	9.0	157.0
	2	[25, 50)	78	3.0	10.0	16.0	21.9	22.0	151.0
	3	[50, 75)	79	13.0	27.5	43.0	59.8	63.0	512.0
	4	[75, 100]	79	27.0	53.5	75.0	112.6	163.5	390.0
2015	1	[0, 25)	78	0.0	1.0	3.0	4.5	6.0	29.0
	2	[25, 50)	79	2.0	9.0	14.0	16.0	19.0	83.0
	3	[50, 75)	79	7.0	20.0	29.0	38.3	41.5	176.0
	4	[75, 100]	79	23.0	38.5	56.0	64.3	78.5	304.0
2016	1	[0, 25)	79	2.0	6.0	12.0	16.9	22.0	67.0
	2	[25, 50)	78	12.0	28.0	37.0	39.5	48.0	91.0
	3	[50, 75)	79	26.0	51.0	60.0	61.7	73.0	92.0
	4	[75, 100]	79	41.0	73.0	85.0	82.2	94.0	100.0

**Fig 6:**
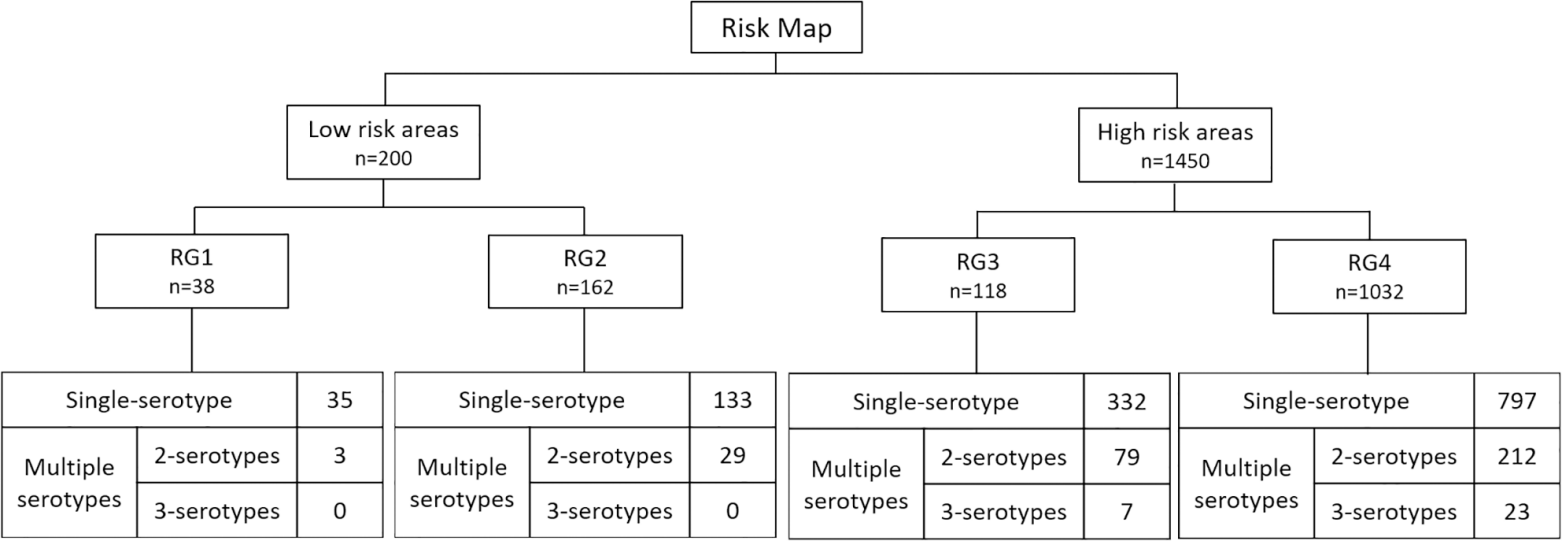
Stratification of clusters (2014–2016) by risk groups and the number of serotypes present in cluster.

Evaluation of risk maps with 2014 to 2016 clusters data shows that the number of dengue clusters in high risk areas was almost 8 times the low risk areas (Fig 6). Each year, close to 90% of the dengue clusters were found in high risk areas, which represent 22% of Singapore land area and 50% of residential areas. The odds of cluster forming in high risk areas was higher than in low risk areas for all three years. The odds ratios were 11.1 (P <0.01), 14.6 (P <0.01) and 12.1 (P <0.01) for 2014, 2015 and 2016 respectively. Clusters were further stratified by the number of serotypes into single serotype and multiple serotypes clusters. High risk areas have a larger proportion of multiple serotypes clusters than low risk areas, and interestingly, 3-serotypes clusters were only present in high risk areas, especially in RG4 (Fig 6). Transmission intensity, comprising of cluster’s growth rate, transmission duration and cluster size were significantly different between single serotype and multiple serotypes clusters (P <0.01). Clusters with more serotypes present have a faster growth rate, longer transmission duration and larger cluster size (Table 3). The same characteristics were seen when we grouped the clusters by high and low risk areas. Though there were less clusters in low risk areas, the transmission intensity of clusters in these low risk areas was of no significant difference (P >0.1) when compared with those in high risk areas (Table 3).

**Table 3:** The median transmission duration, cluster size and growth rate of clusters with different number of serotypes present in Singapore (2014–2016), and separated by low^a^ and high^b^ risk areas.

No. of serotypes in cluster	Median transmission duration (days)			Median cluster size (no. of cases)			Median growth rate (no. of new cases per day)			No. of clusters		
	Combined*	Low risk areas	High risk areas	Combined*	Low risk areas	High risk areas	Combined*	Low risk areas	High risk areas	Combined	Low risk areas	High risk areas
1	13	13	13	4	4	4	0.28	0.28	0.28	1319	168	1129
2	21	18	21	7	7	7	0.35	0.34	0.35	328	32	291
3	53	n/a	93	25	n/a	70	0.52	n/a	0.68	31	0	30
4	n/a	n/a	n/a	n/a	n/a	n/a	n/a	n/a	n/a	0	0	0

^a^ Low risk areas are RG 1 and RG 2 grids.

^b^ High risk areas are RG 3 and RG 4 grids.

n/a, data not available

Kruskal-Wallis test (P <0.01 are highlighted by *)

## Discussion

Dengue has been endemic in Singapore since its first reported outbreak in 1901 [26]. Though the dengue temporal model is capable of predicting impending outbreaks, it does not indicate where the outbreak will be [17]. As a result, source reduction inspections are conducted on a frequency based on the risk level of the premises types (e.g. construction sites are of higher risk than apartment homes). Spatial risk mapping of dengue transmission is therefore essential for the prioritization and allocation of scarce resources especially manpower need to inspect premises.

Dengue risk map has been developed in many countries as a surveillance tool to enhance public health preparedness for dengue outbreak [27]. Statistical approaches such as logistic regression models, generalized linear models and general additive models were most commonly used to compute risk level and create dengue risk map [28–32]. Although very good predictive accuracy can be achieved from Random Forest, it has yet to be reported in the development of dengue risk map [27]. In this paper, we demonstrated the use of Random Forest, an ensemble learning method that has garnered much interest in the machine-learning community, to develop a dengue risk map with high accuracy and robustness. Studies have shown that Random Forest has excellent performance in classification tasks, and even outperforms its counterparts such as discriminant analysis, neural networks and support vector machines [33,34]. The methodology has several advantages over the traditional approaches, with the utmost advantage being highly tolerant to interactions among the input covariates. Dengue transmission is a multi-factorial stochastic process where often one risk factor is correlated with other risk factors, making it difficult to quantify the effect of a particular risk factor as well as to construct a risk map using classical modelling method such as regression.

The model ranked the overall risk of dengue transmission of different areas in a year and mapped the ranks as color-coded risk groups. By comparing the risk groupings of the grids over the years, NEA could identify recurring risk areas (i.e. grids that are persistently risk group 4 over the years) that are of concerns, fluctuating risk areas (i.e. grids that have fluctuating risk grouping over the years) and even potential risk areas that were not seen in the previous years (i.e. grids whose risk group change from 1 to 4). Evaluation using latest dengue case data showed the model had strong predictive capability. Strong positive correlation between the observed and predicted risk ranks, and an almost perfect agreement between the predicted risk levels and case density were observed. High risk areas are where clusters, in particular multiple serotypes clusters are most likely to occur. However, surprisingly, despite the difference in risk levels, there was no difference in the transmission intensity of clusters in high and low risk areas, and this may be attributed to the presence of small pockets of high *Ae*. *aegypti* population within the low risk areas. For instance, construction sites along Flora Road and Belgravia Drive had led to large dengue cluster of size 46 and 35 in traditionally low risk areas in 2014 and 2016 respectively. This, therefore, highlights the importance of ground inspections in identifying high risk sites in low risk areas.

The dengue risk map complements the dengue temporal model in allowing the operation department of NEA to prioritise vector control efforts. While the dengue temporal model provides the time component of when the next outbreak will be, it is thus now possible for NEA to deploy limited resources ahead of time, targeting at the places with high risk of transmission.

There are, however, some limitations to the use of Random Forest, the key on being the model not amenable to interpretation. The Random Forest is an ensemble method―it constructs many “weak” models and then combines them to achieve a “strong” model. There is no explicit formulae-form relationship between risk of dengue transmission and risk factors, making it virtually impossible to decompose a particular prediction output into contribution of risk factors. Understanding that the primary objective is to accurately stratify the risk of dengue transmission liberated us from concerns over interpretability. Nevertheless, the Random Forest model is able to offer some insights about dengue transmission by estimating importance and partial effects of variable at a macro level.

The dengue risk map has become an integral part of Singapore’s dengue control program. The dengue risk map would be generated at the start of each year, and NEA operations would use the risk map as a guide to prioritize resource allocation for dengue control and plan the preventive surveillance activities for the year. Dengue risk map has been used since 2015 by the operational division of NEA to guide targeted preventive interventions. Future work will include incorporating real time data to develop a spatio-temporal risk map.

### Conclusions

This study demonstrates the potential of Random Forest and its strong predictive capability in stratifying the spatial risk of dengue transmission in Singapore. Dengue risk map produced using Random Forest has high accuracy, and is a good tool to guide vector control operations, allowing targeted preventive measures before and in times of dengue outbreak. Valuable resources can then be deployed in a strategic manner, mitigating the spread of dengue transmission.
